# Sensory-Analytical Comparison of the Aroma of Different Horseradish Varieties (*Armoracia rusticana*)

**DOI:** 10.3389/fchem.2018.00149

**Published:** 2018-05-07

**Authors:** Eva-Maria Kroener, Andrea Buettner

**Affiliations:** ^1^Chair of Aroma and Smell Research, Department of Chemistry and Pharmacy, Emil Fischer Center, Friedrich-Alexander-Universität Erlangen-Nürnberg, Erlangen, Germany; ^2^Department of Sensory Analytics, Fraunhofer Institute for Process Engineering and Packaging IVV, Freising, Germany

**Keywords:** isothiocyanates, pungency, semi-quantitative analysis, allyl isothiocyanate, 2-phenylethyl isothiocyanate, 3-isopropyl-2-methoxypyrazine, wine lactone

## Abstract

Horseradish (*Armoracia rusticana*) is consumed and valued for the characteristic spicy aroma of its roots in many countries all over the world. In our present study we compare six different horseradish varieties that were grown under comparable conditions, with regard to their aroma profiles, using combined sensory-analytical methods. Horseradish extracts were analyzed through gas chromatography-olfactometry (GC-O) and their aroma-active compounds ranked according to their smell potency using the concept of aroma extract dilution analysis (AEDA). Identification was carried out through comparison of retention indices, odor qualities and mass spectra with those of reference substances. Besides some differences in relative ratios, we observed some main odorants that were common to all varieties such as 3-isopropyl-2-methoxypyrazine and allyl isothiocyanate, but also characteristics for specific varieties such as higher contents for 3-isopropyl-2-methoxypyrazine in variety Nyehemes. Moreover, three odorous compounds were detected that have not been described in horseradish roots before.

## Introduction

**Figure 1:**
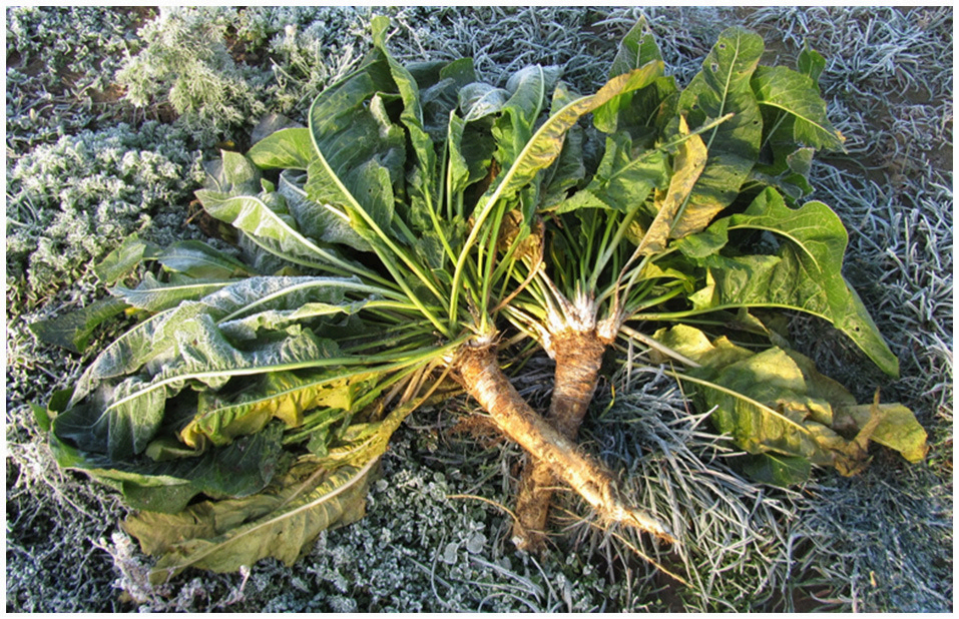
Representative photograph of harvested horseradish plants.

Horseradish (Figure 1) is a hardy perennial plant (Weber, 1949) that is cultivated and consumed in many parts of the world. It is also known by its botanical name *Armoracia rusticana* (Gaertn., Mey. et Scherb.) and belongs to the family of Brassicaceae (Shehata et al., 2009), that includes other well-known plants and vegetables like broccoli, cabbage, rapeseed and wasabi. Of particular importance for the industry are the main roots of horseradish plants, as they are processed to tasty condiments (Shehata et al., 2009). However, horseradish also enjoys a good reputation as medicinal plant in naturopathy both in the past and present. Horseradish roots have been reported to support and strengthen the body's defenses, due to their high natural vitamin C content, and have been used as medicine for scurvy in the past (Bladh and Olsson, 2011). They are further administered as a treatment for respiratory tract infections like coughs, bronchitis and sinuses, and urinary tract infections, as well as a remedy for headaches and pain associated with rheumatism (Schulz, 2008; Sampliner and Miller, 2009; Sarli et al., 2012).

Those positive effects on human health are supported by a series of studies. Horseradish extract has been shown to inhibit gram-positive and gram-negative bacteria, like *Bacillus subtilis, Escherichia coli, Staphylococcus aureus* (Halbeisen, 1957; Kienholz and Kemkes, 1960) and shows antimicrobial activity against *Mycobacterium tuberculosis*, the causal agent of tuberculosis (Kemkes and Kienholz, 1957). *In vitro* experiments also demonstrated horseradish root extracts to show antioxidative, peroxidase and radical scavenging activities, as well as antimutagenic activity with respect to 2-amino-3,8-dimethylimidazo[4,5-f]quinoxaline (MeIQx) and 3-chloro-4-(dichloromethyl)-5-hydroxy-5*H*-furan-2-one (MX) (Kinae et al., 2000). Horseradish extract further shows antifungal activities against *Sclerotium rolfsii* Sacc., *Fusarium oxysporum* Schlecht. and *Fusarium culmorum* (Wm.G.Sm.) Sacc., which are pathogenic fungi that cause extensive agricultural losses (Tedeschi et al., 2011), as well as fumigant activity against several insect pests, like the Indian mealmoth (*Plodia interpunctella*), the maize weevil (*Sitophilus zeamais*) and the Asian tiger mosquito (*Aedes albopictus*) (Chen et al., 2011; Tedeschi et al., 2011).

The organic isothiocyanates (ITCs) allyl isothiocyanate (AITC) and 2-phenylethyl isothiocyanate (PEITC) are enzymatically formed upon rupture of horseradish root cells and represent, on a quantitative basis, the major constituents of the essential oil of horseradish roots (Stoll and Seebeck, 1948; Isaac and Kohlstaedt, 1962; Gilbert and Nursten, 1972). AITC and PEITC both showed high antimicrobial activities against *Bacillus subtilis, Escherichia coli, Staphylococcus aureus, Candida albicans, Pseudomonas* aeruginosa, as well as an antifungal activity against *Aspergillus niger* (Masuda et al., 1999). AITC has further been proven to exhibit bactericidal effects against *Mycobacterium tuberculosis* (Foter, 1940) and PEITC has been demonstrated to be an effective inhibitor of nitrosamine-induced tumorigenesis (Stoner and Morse, 1997). Both isothiocyanates have also been proven to be effective insecticides against the house fly (*Musca domestica*), PEITC additionally against the vinegar fly (*Drosophila melanogaster*) and AITC against the lesser grain borer (*Rhizopertha dominica*) (Lichtenstein et al., 1962; Peterson et al., 1998). Moreover, those two substances also play an important role in the overall sensory properties of horseradish roots, triggering pungency besides their characteristic horseradish-like aroma impression; due to this bi-sensory effect, namely trigeminal and aroma, horseradish roots and their products are valued as a condiment in daily cuisine (Gilbert and Nursten, 1972; Masuda et al., 1996; Kroener and Buettner, 2017). However ITCs are not the only chemosensorially active compound class in horseradish. Besides other odorous ITCs like *sec*-butyl ITC, benzyl ITC and 3-(methylthio)propyl ITC, we recently reported additional aroma-active substances belonging to other structural groups: amongst others pyrazines, like 3-isopropyl-2-methoxypyrazine and 3-*sec*-butyl-2-methoxypyrazine, aliphatic acids, like acetic acid and butanoic acid, and fatty acid-derived carbonyl compounds, like *(Z)*-3-hexenal and 1-octen-3-one (Kroener and Buettner, 2017). Other detected odorous compounds were for example (3*S*,3a*S*,7a*R*)-3,6-dimethyl-3a,4,5,7a-tetrahydro-3*H*-1-benzofuran-2-one ((3*S*,3a*S*,7a*R*)-wine lactone), 1-cyano-2,3-epithiopropane (CETP) and 3-methylindole (skatole). Based on their high flavor dilution (FD) factors it was assumed that 3-isopropyl-2-methoxypyrazine, (3*S*,3a*S*,7a*R*)-wine lactone, AITC and PEITC are amongst those aroma compounds which most likely contribute significantly to the general aroma impression of freshly grated horseradish roots. Our previous study, however, comprised six varieties, having been grown on different soils and during different periods of cultivation. Accordingly, these studies do not allow for direct comparison between varieties.

In our present investigation we therefore aimed at expanding our knowledge by directly comparing different horseradish varieties with regard to their aroma composition. To this aim, the selected varieties, namely VFS × BA, BA × VFS, BA, Kroener, Nyehemes and NA, were grown on the same acreage within the same time period to exclude different environmental influences, and were harvested under identical conditions. General differences and similarities should then be elaborated on a relative basis comprising comparative aroma extract dilution analyses (Buettner and Schieberle, 2001).

## Materials and methods

### Horseradish samples

As in our previous study we selected the horseradish varieties VFS × BA, BA × VFS, BA, Kroener, Nyehemes, and NA (Kroener and Buettner, 2017). Varieties BA and Nyehemes are of Hungarian origin. Varieties VFS × BA and BA × VFS were bred through generative propagation between variety BA and the Hungarian variety VFS. For VFS × BA the mother plant is VFS, for BA × VFS the mother plant is BA. Variety Kroener is a regionally wide spread variety that originally comes from Austria. Variety NA originates from American horseradish. Sprouted sets of all varieties, gained by vegetative reproduction, were planted on the same field near the city of Baiersdorf, Germany, in April 2015, and were grown under the same growing conditions for the same period of time. To promote accurate growth and sizing of the roots, the sets were coated with a loose plastic foil from the crown end to about half of its size to avoid growth of secondary roots in the upper half of the sets. Fertilizer was applied following local practice: K_2_O 200 kg/ha, N 200 kg/ha and P_2_O_5_ 70 kg/ha. Agricultural pesticides ware applied following local practices as well. The horseradish plants were harvested in November of the same year. One main root from each variety was selected for investigation. Roots were chosen to represent about the same length (approximately 25–30 cm) and weight (approximately 570–750 g).

### Chemicals

Isobutyl ITC 97%, 4-pentenyl ITC 95% and *sec*-butyl ITC 97% were purchased from ABCR (Karlsruhe, Germany), 3-*sec*-butyl-2-methoxypyrazine from Acros Organics (Geel, Belgium), (3*S*,3a*S*,7a*R*)-wine lactone >95% from Aromalab (Freising, Germany), 2-(methylthio)ethyl ITC >96% from FCH Group (Chernigiv, Ukraine), butanoic acid ≥99% from Fluka (Steinheim, Germany), *(Z)*-3-hexenal 50% from SAFC (Steinheim, Germany) and 3-methylbutyl ITC >98% from Santa Cruz Biotechnology (Heidelberg, Germany). Acetic acid >99%, AITC 95%, *p*-anisaldehyde 98%, benzyl ITC 98%, 3-hydroxy-4,5-dimethyl-2(5*H*)-furanone (sotolone) ≥97%, 3-isobutyl-2-methoxypyrazine ≥99%, 3-isopropyl-2-methoxypyrazine 99%, 2-methylbutanoic acid 98%, 3-methylbutanoic acid 99%, skatole 98%, 3-(methylthio)propanal 98%, 3-(methylthio)propyl ITC 98%, 1-octen-3-one 50%, 2-phenylacetaldehyde 90% and PEITC >99% were purchased from Sigma-Aldrich (Steinheim, Germany) and 3-butenyl ITC 95% from TCI Europe (Eschborn, Germany). CETP was synthesized as described in our previous study (Kroener and Buettner, 2017).

Dichloromethane (DCM), which was freshly distilled for purification prior to use, and anhydrous sodium sulfate were purchased from VWR International GmbH (Darmstadt, Germany).

### Sample preparation

The selected main roots of each horseradish variety were peeled and a small piece with a length of about 5 cm of the center was ground into small pieces of about 1 mm^3^ with a Moulinex Moulinette (Krups GmbH, Frankfurt am Main, Germany). One gram of the ground sample material was immediately applied for solvent extraction with 30 ml DCM under vigorous stirring for 30 min. The extracts were subsequently filtrated and dried over anhydrous sodium sulfate, then purified via solvent-assisted flavor evaporation (SAFE) at 50°C (Engel et al., 1999). The obtained distillates were subjected to Vigreux distillation and micro-distillation (Bemelmans, 1979), being thereby reduced to a final volume of 100 μl.

### High resolution gas chromatography-olfactometry

HRGC-O was performed with a GC type Trace Ultra (Thermo Finnigan, Dreieich, Germany) using either a DB-FFAP (30 m × 0.32 mm, film thickness 0.25 μm; J&W Scientific) or a DB-5 (30 m × 0.32 mm, film thickness 0.25 μm; J&W Scientific, Finsons Instruments, Mainz-Kastel, Germany) capillary column.

The injection of the samples was carried out at 40°C via the cold on-column application technique. After holding this temperature for 2 min, the temperature was raised at 8°C/min to 240°C in case of DB-FFAP, and to 200°C in case of DB-5 column. For DB-FFAP, the 240°C were held for 5 min, whereas for DB-5 the temperature was additionally increased at 40°C/min to 300°C, and finally held for 5 min. The flow of the helium carrier gas was held constant at 2.5 ml/min. The carrier gas flow was split using a Y-splitter at the end of the main capillary column in a ratio 1:1. Two equally long deactivated capillary columns were connected to the Y-splitter that led the separated gas stream to a sniffing port and a flame ionization detector (FID), both kept at 250°C. The injection volumes for all samples and solutions were 2 μl each.

Comparison of the retention indices (RI) (van Den Dool and Kratz, 1963), the odor qualities and odor intensities on both capillary columns with those of authentic reference standards led to the tentative identification of a series of aroma-active compounds in the horseradish samples, being then further substantiated by mass spectrometric analyses as described below.

### Aroma extract dilution analysis

An aroma extract dilution analysis (AEDA) was conducted as described by Grosch (1993). Aliquots of the concentrated horseradish extracts (total volume 100 μl) were diluted stepwise 1+1 (v/v) with DCM. The original extracts (FD = 1) and the diluted samples (FD = 2, 4, 8, etc.) were then successively analyzed by HRGC-O (see section High resolution gas chromatography-olfactometry) until no more odor could be sensorially perceived. The resulting flavor dilution (FD) factors of the odorants were determined on capillary column DB-5. The sniffing analyses were performed by one main, trained investigator and cross-checked by two other trained panelists on selected samples. To ascertain, if the original extracts (FD = 1) represent the original aroma of the samples, they were additionally directly smelled at.

### High resolution gas chromatography-mass spectrometry

HRGC-MS was conducted using a 7890A GC (Agilent Technologies, Santa Clara) and a 5975C inert XL EI/CI MSD mass spectrometer (Agilent Technologies), either on a capillary column DB-FFAP (30 m × 0.25 mm, film thickness 0.25 μm; J&W Scientific) or on a DB-5 column (30 m × 0.25 mm, film thickness 0.25 μm; J&W Scientific). Temperature programs and the application technique were the same as described in section High resolution gas chromatography-olfactometry, with the sole exception that on DB-5 the second heating rate was 20°C/min instead of 40°C/min. The flow rate of the carrier gas helium was 1.2 ml/min and the injection volume was 1 μl each. EI-mass spectra were generated in full scan mode with an m/z range from 40 to 400 at 70 eV ionization energy.

### Two-dimensional high resolution gas chromatography-mass spectrometry/olfactometry (HRGC-GC-MS/O)

HRGC-GC-MS/O was performed with two coupled CP 3800 GCs (Varian, Darmstadt, Germany) that were linked through a cryogenic trapping system CTS 1 (Gerstel, Mülheim an der Ruhr, Germany), in combination with a Saturn 2200 MS (Varian), which was connected to the second GC. The first GC was additionally equipped with a multi-column switching system MCS 2 (Gerstel). Capillary columns used were a DB-5 in the first oven and a DB-FFAP in the second oven, and vice versa if necessary due to specific coelution issues. The capillary column dimensions in the first oven were 30 m × 0.32 mm with film thickness 0.25 μm (J&W Scientific) and in the second oven 30 m × 0.25 mm with film thickness 0.25 μm (J&W Scientific). The flow rate of the helium carrier gas was 2.0 ml/min. The gas flow was split between a sniffing port and an FID (first oven) or MS (second oven) at the end of the main capillary column in each oven. The sniffing ports and the FID were kept at 260° and 240°C, respectively. The injection was conducted in the first oven at 40°C using 2 μl sample or reference solutions. The initial temperature of 40°C was held for 2 min, afterwards increased at 8°C/min to 240°C, and finally held for 5 min. After transfer of the target compounds from the first oven, the temperature of the second oven was immediately raised from 40° to 250°C (240°C for DB-FFAP) at 8°C/min, and then held for 5 min. EI-mass spectra were generated in full scan mode with an m/z range from 40 to 250 at 70 eV ionization energy.

Multi-trapping was required in several cases to receive adequate enrichment of the target substances and to achieve the necessary match with the spectra of the corresponding reference compounds. In this case, the same substance was cut in each case in the course of four up to seven runs and collected in the cryogenic trap; then, the combined cuts from these runs were transferred onto the second capillary column in the second oven at once. Accordingly, an increased total amount of the target compound was gained for mass spectrometric detection.

### Semi-quantitative analysis of the most important aroma compounds

The content of those horseradish aroma compounds, which are most likely to have the greatest influence on the overall aroma due to their high FD factors, were semi-quantitatively estimated in the respective roots on a comparative basis using external standards. To this aim, standard solutions of each corresponding reference compound were prepared with approx. the same concentration as the target compound in the analyzed samples using FID analysis or a concentration of approximately 2 μg/ml using MS analysis, respectively, as will be detailed in the following. Analyses were carried out on each of the concentrated horseradish extracts of the different varieties (see section Sample preparation). The relative content of AITC, benzyl ITC, *sec*-butyl ITC, CETP, *(Z)*-3-hexenal, 3-(methylthio)propyl ITC and PEITC was determined by HRGC-O/FID (see section High resolution gas chromatography-olfactometry) on basis of a direct comparison of the peak areas of each sample analysis and the respective standard FID chromatograms. In case of 3-isopropyl-2-methoxypyrazine, *sec*-butyl-2-methoxypyrazine and skatole, the content was determined by HRGC-MS (see section High resolution gas chromatography-mass spectrometry), and in case of (3*S*,3a*S*,7a*R*)-wine lactone by HRGC-GC-MS/O (see section Two-dimensional high resolution gas chromatography-mass spectrometry/olfactometry (HRGC-GC-MS/O)). The respective peak areas of the following m/z values served as basis for the calculation of the relative concentration levels: m/z = 137 for 3-isopropyl-2-methoxypyrazine, m/z = 138 for 3-*sec*-butyl-2-methoxypyrazine, m/z = 130 for skatole and m/z = 151 for (3*S*,3a*S*,7a*R*)-wine lactone.

## Results

A total of 39 odor-active compounds was successfully detected by HRGC-O; thereby, eight compounds were tentatively identified based on a comparison of their retention indices (van Den Dool and Kratz, 1963) and their sensory characteristics with those of authentic reference standards, whereas 18 compounds were identified based on these criteria, and additionally via comparison of their mass spectrometric data (cf. Table 1). Accordingly, all substances with FD ≥ 32 could be successfully related to their respective reference substances, with the sole exception of the unknown substances at RI 1011 and RI 1214. These substances exhibited FD factors of 32 and 64 respectively in varieties VFS × BA and/or Nyehemes, but could not be assigned to any compound in our comprehensive database, and did not provide any interpretable mass spectrum that allowed for tentative identification. Apart from that, the identity of another 11 aroma-active components (no. 2, 3, 12, 18, 20, 23, 26, 30, 32, 33, and 36 cf. Table 1) remained unresolved; however, their FD factors did not exceed values of 16 in any case, and 10 of these compounds were, moreover, only detectable in one horseradish sample in each case. Of these, the vinegar-like, spicy, fermented smelling substance with an RI of 1235 in the variety NA and the substance with a grape juice-like odor and an RI of 1372 in the variety Kroener were detected with FD factors of 16 and 8, respectively. Two unknown substances were perceivable in all horseradish varieties; the before mentioned substance at RI 1011 with a vinegar- and cabbage-like smell (FD 4-64), and the substance at RI 1053 with an earthy, dusty, metallic smell (FD 1-16). The before mentioned unknown compound with an RI of 1214 and a fatty, spicy, onion-, cabbage- and garlic-like odor impression (FD 2-32) was found in all varieties except VFS × BA. As mentioned in our previous study, we assume that the unidentified compound at RI 1053 might be an alkylated pyrazine and the unknown substances with a vinegar-, cabbage-, garlic-like or onion-like odor could be sulfur-containing molecules, due to sensory similarities (Kroener and Buettner, 2017).

**Table 1:** Comparison of aroma compounds in six different horseradish varieties by means of AEDA on DCM solvent extracts of the main roots.

**No. ^a^**	**Odorant^b^**	**Odor quality^c^**	**RI value^d^ on**		**FD factor^e^**					
			**DB-5**	**DB-FFAP**	**VFS × BA**	**BA × VFS**	**BA**	**Kroener**	**Nyehemes**	**NA**
1	Acetic acid^g^	Vinegar-like	633	1,452	2	4	2	4	1	1
2	Unknown	Fatty	756	nd ^h^	nd	nd	4	nd	nd	nd
3	Unknown	Pungent, onion-like	775	nd ^h^	nd	nd	nd	2	nd	nd
4	*(Z)*-3-Hexenal^g^	Grassy, green	807	1,149	512	128	32	128	128	128
5	Butanoic acid^f^	Cheesy	800	1,629	nd	nd	4	2	nd	nd
6	3-Methylbutanoic acid^f^	Cheesy	854	1,671	nd	nd	nd	4	1	4
7	2-Methylbutanoic acid^f^	Cheesy	868	1,670	2	1	4	4	4	1
8	Allyl isothiocyanate^g^	Pungent, mustard-like, horseradish-like, onion-like	884	1,361	2,048	1,024	2,048	2,048	2,048	2,048
9	3-(Methylthio)propanal^f^	Cooked potato-like	911	1,458	32	8	16	16	32	32
10	*sec*-Butyl isothiocyanate^g^	Pungent, chemical, green	933	1,269	16	8	16	32	8	8
11	Isobutyl isothiocyanate^g^	Pungent	955	1,323	1	1	1	1	2	1
12	Unknown	Fruity, acidic	969	nd^h^	nd	nd	nd	nd	2	nd
13	1-Octen-3-one^f^	Mushroom-like	978	1,311	4	4	4	2	4	4
14	3-Butenyl isothiocyanate^g^	Pungent	981	1,454	4	4	4	4	8	4
15	1-Cyano-2,3-epithiopropane^g^	Onion-like, pungent, fatty, spicy	999	1,833	32	<1^i^	32	32	16	<1^i^
16	Unknown	Vinegar-like, cabbage-like	1,011	nd^h^	64	16	8	4	32	16
17	2-Phenylacetaldehyde^g^	Honey-like, sweet	1,043	1,646	4	4	32	16	8	8
18	Unknown	Earthy, dusty, metallic	1,053	nd^h^	2	2	1	16	8	4
19	3-Methylbutyl isothiocyanate^g^	Pungent	1,059	1,425	<1^i^	1	1	1	1	1
20	Unknown	Fatty, vinegar-like, onion-like	1,068	nd ^h^	nd	nd	2	nd	nd	nd
21	4-Pentenyl isothiocyanate^g^	Pungent	1,082	1,536	2	4	2	4	4	4
22	3-Isopropyl-2-methoxypyrazine^g^	Green pepper-like	1,093	1,427	2,048	2,048	1,024	1,024	8,192	4,096
23	Unknown	Cabbage-like, vinegar-like	1,105	nd^h^	nd	nd	nd	nd	2	nd
24	3-Hydroxy-4,5-dimethyl-2(5*H*)-furanone (sotolone)^f^	Spicy, lovage-like	1,120	2,203	nd	nd	8	4	nd	4
25	3-*sec*-Butyl-2-methoxypyrazine^g^	Green pepper-like	1,171	1498	32	64	128	128	256	64
26	Unknown	Fatty, earthy	1,178	nd ^h^	nd	nd	nd	1	nd	nd
27	3-Isobutyl-2-methoxypyrazine^f^	Sweet bell pepper-like	1,182	1,523	16	8	2	8	4	32
28	2-(Methylthio)ethyl isothiocyanate^g^	Pungent	1,203	1,900	<1^i^	<1^i^	<1^i^	<1^i^	2	<1^i^
29	Unknown	Fatty, spicy, onion-like, cabbage-like, garlic-like	1,214	nd^h^	nd	8	2	4	32	4
30	Unknown	Vinegar-like, fermented, spicy	1,235	nd^h^	nd	nd	nd	nd	nd	16
31	*p*-Anisaldehyde^f^	Sweet woodruff-like	1,257	2,029	4	4	4	4	4	4
32	Unknown	Vinegar-like, onion-like	1,276	nd^h^	2	nd	nd	nd	nd	nd
33	Unknown	Pungent, vinegar-like	1,297	nd^h^	nd	nd	nd	nd	nd	1
34	3-(Methylthio)propyl isothiocyanate^g^	Mushroom-like	1,310	1,983	8	8	32	16	32	32
35	Benzyl isothiocyanate^g^	Pungent, watercress-like	1,365	2,097	16	32	16	16	16	16
36	Unknown	Grape juice-like	1,372	nd^h^	nd	nd	nd	8	nd	nd
37	3-Methylindole (skatole)^g^	Fecal	1,389	2,502	32	16	64	64	64	64
38	(3*S*,3a*S*,7a*R*)-Wine lactone^g^	Sweet, smoky, peach-like, coconut-like	1,461	2,232	1,024	1,024	1,024	512	1,024	2,048
39	2-Phenylethyl isothiocyanate^g^	Horseradish-like, pungent, watercress-like	1,465	2,222	64	512	512	256	512	2,048

*Displayed are the FD factors and the odor qualities of the compounds sorted by RI values on DB-5 capillary column*.

a *Numbers correspond to Figure 3*.

b *The compounds were identified by comparing them with the reference odorant based on the given criteria (see below)*.

c *Odor quality as perceived at the sniffing port*.

d *Retention indices according to van Den Dool and Kratz (1963)*.

e *Flavor dilution (FD) factor on the capillary column DB-5*.

f *Identification criteria: RIs on capillaries named in table, odor quality and intensity at the sniffing port*.

g *Identification criteria: same as in (f) and MS-EI data*.

h *nd, not determined (a) due to unsatisfactory separation on this analytical column or (b) inconclusive assignment of the smell to a specific retention factor due to co-elution with other odor-active substances*.

i *Detected via HRGC-MS, but due to contents under the odor threshold not olfactorily perceivable*.

**Figure 2:**
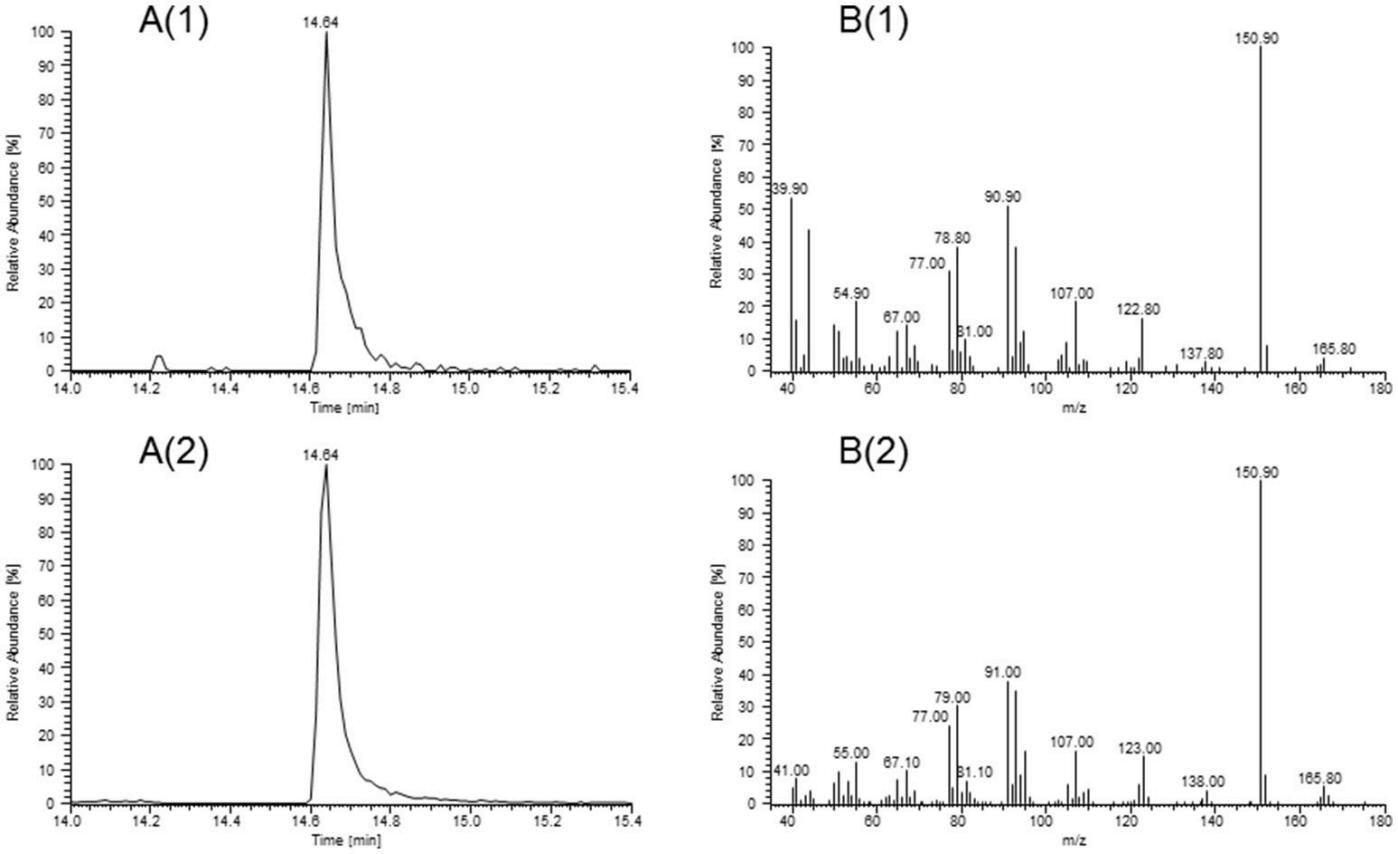
Identification of (3*S*,3a*S*,7a*R*)-wine lactone in horseradish by HRGC-GC-MS/O. Extracted ion chromatograms at m/z = 151 **(A)** and mass spectra **(B)** of (3*S*,3a*S*,7a*R*)-wine lactone in the sample of variety BA (1) and of the corresponding reference standard (2).

Substances found with overall high FD factors in all samples were the pungent, mustard-, horseradish- and onion-like smelling AITC (FD 1024-2048), the horseradish-like, pungent, watercress-like smelling PEITC (FD 64-2048), the grassy, green smelling *(Z)*-3-hexenal (FD 32-512), the green pepper-like smelling pyrazines 3-isopropyl-2-methoxypyrazine (FD 1024-8192) and 3-*sec*-butyl-2-methoxypyrazine (FD 32-256). Apart from that, we detected skatole (FD 16-64), which gives a fecal odor impression, and the sweet, smoky, peach- and coconut-like smelling (3*S*,3a*S*,7a*R*)-wine lactone (FD 512-2048). In our previous study (Kroener and Buettner, 2017), (3*S*,3a*S*,7a*R*)-wine lactone could only be tentatively identified, whereas in this study we were now able to obtain a mass spectrum of this compound which matched with the MS spectrum of the (3*S*,3a*S*,7a*R*)-wine lactone standard (Figure 2).

Of the group of ITCs we further successfully confirmed the pungent, chemical, green smelling *sec*-butyl ITC (FD 8-32), the pungent ITCs isobutyl ITC (FD 1-2), 3-methylbutyl ITC (FD <1-1), 3-butenyl ITC (FD 4-8), 4-pentenyl ITC (FD 2-4), 2-(methylthio)ethyl ITC (FD <1-2), the pungent, watercress-like smelling benzyl ITC (FD 16-32) and 3-(methylthio)propyl ITC (FD 8-32), with a mushroom-like odor quality.

The only odor active nitrile found was CETP. This substance, however, showed a pronounced variation in FD factors between the different varieties investigated in this study, ranging from FD <1 to 32.

Of the group of aliphatic acids the vinegar-like smelling acetic acid (FD 1-4) and the cheesy smelling acids butanoic acid (nd - FD 4), 2-methylbutanoic acid (FD 1-4) and 3-methylbutanoic acid (nd - FD 4) could be detected. Other odor-active substances were the mushroom-like smelling 1-octen-3-one (FD 2-4), the cooked potato-like smelling 3-(methylthio)propanal (FD 8-32), the honey-like, sweet smelling 2-phenylacetaldehyde (FD 4-32), the sweet woodruff-like smelling *p*-anisaldehyde (FD 4) and the spicy, lovage-like smelling sotolone (nd - FD 8). Another pyrazine found was 3-isobutyl-2-methoxypyrazine (FD 2-32) with a sweet bell pepper-like odor.

The main aim of our study was to compare different horseradish varieties to elaborate common aroma features, and characteristics of each variety. Thereby, it became evident that the variety Nyehemes stood out to some extent, as it had the highest FD factors for 3-isopropyl-2-methoxypyrazine (FD 8192) and 3-*sec*-butyl-2-methoxypyrazine (FD 256) and at the same time one of the lowest for 3-isobutyl-2-methoxypyrazine (FD 4). It also showed the highest FD factor for the unknown compound with a fatty, spicy, onion-, cabbage- and garlic-like smell at RI 1214 (FD 32).

CETP was olfactorily undetectable in the varieties BA × VFS and NA, whereas relevant FD factors were observed for this substance in the other varieties (FD 16-32). BA × VFS further showed a slightly higher FD factor for benzyl ITC (FD 32), but marginally lower factors for AITC (FD 1024) and 3-(methylthio)propanal (FD 8) than the other varieties. The variety NA, on the other hand, yielded the highest FD factors for PEITC (FD 2048), (3*S*,3a*S*,7a*R*)-wine lactone (FD 2048) and 3-isobutyl-2-methoxypyrazine (FD 32). The by far lowest FD factor was recorded for the variety VFS × BA in case of PEITC (FD 64) and for 3-*sec*-butyl-2-methoxypyrazine (FD 32), whereas the highest FD factors were found in the same variety for *(Z)*-3-hexenal (FD 512) for the unknown substance with vinegar- and cabbage-like odor at RI 1011 (FD 64). In contrast to that, the lowest FD factor for *(Z)*-3-hexenal (FD 32) was determined for the variety BA, together with the lowest FD factors for 3-isobutyl-2-methoxypyrazine (FD 2) and the earthy, dusty, metallic smelling unknown compound at RI 1053 (FD 1). With regards to the latter compound, the highest FD factor (FD 16) was found for the variety Kroener, together with the highest FD factor for *sec*-butyl ITC (FD 32), whereas this variety was characterized by the lowest factor for the unknown substance at RI 1011 (FD 4) and a slightly lower value for PEITC (FD 512) than those determined for the other varieties.

**Figure 3:**
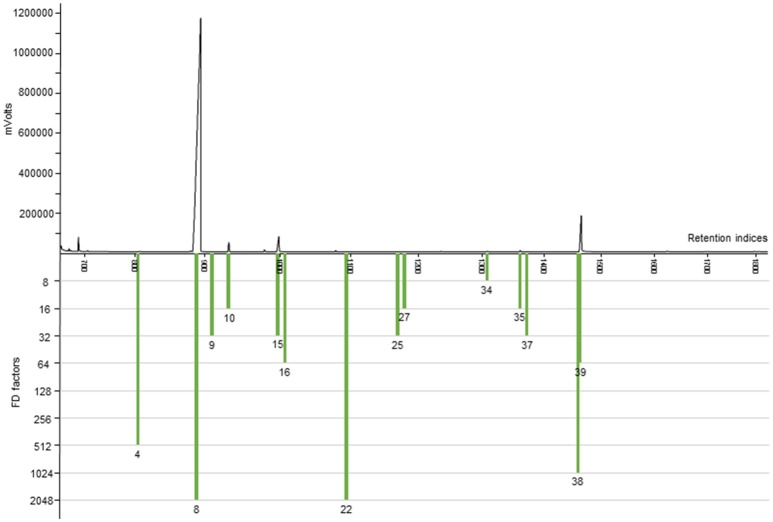
HRGC-O/FID chromatogram of a diluted horseradish root extract (FD 8) of variety VFS × BA. Presented are the aroma-active substances with an FD factor ≥8. Numbers correspond to Table 1.

To further substantiate our relative comparative findings that were based on GC-O, the content of 11 important and representative aroma-active substances in horseradish roots of six different horseradish varieties was estimated via semi-quantitative analyses (cf. Tables 2, 3). The highest contents by far were found for AITC (936–1,990 μg/g). A representative FID chromatogram with the example of variety VFS × BA is shown in Figure 3, together with its FD factors given in Table 1. The chromatogram exemplarily displays the prominent peak of AITC that stands out amongst the other volatile constituents of the horseradish aroma. These abundant values are followed by those of PEITC (approximately 40–270 μg/g) and CETP, the latter showing an exceptionally wide range between minimum and maximum values of 0.2–25.1 μg/g, thereby spanning two orders of magnitude between extremes. In the lower μg/g-range we detected *sec*-butyl ITC (3.7–8.7 μg/g), *(Z)*-3-hexenal (0.7–5.1 μg/g), 3-(methylthio)propyl ITC (0.8–4.6 μg/g) and benzyl ITC (0.8–2.1 μg/g). The four remaining aroma-active substances showed contents in the ng/g-range and, accordingly, much lower contents than the aforementioned substances. Thereby, the values roughly ranged between about 10 and 100 ng/g in case of 3-isopropyl-2-methoxypyrazine (6.8–95.2 ng/g) and 3-*sec*-butyl-2-methoxypyrazine (6.7–58.1 ng/g), whereas the contents of skatole and (3*S*,3a*S*,7a*R*)-wine lactone were in the low ng/g-range with 1.3 to 13.2 ng/g and 1.0–10.2 ng/g, respectively.

**Table 2:** Content [μg/g] of important aroma-active compounds in one gram of freshly ground horseradish root of six different horseradish varieties, determined by HRGC-O/FID.

**Odorant**	**Content [μg/g]**					
	**VFS x BA**	**BA × VFS**	**BA**	**Kroener**	**Nyehemes**	**NA**
Allyl isothiocyanate (AITC)	1110	936	1430	1490	1990	1420
2-Phenylethyl isothiocyanate (PEITC)	39.6	242	124	64.7	153	270
1-Cyano-2,3-epithiopropane (CETP)	24.9	0.2	15.9	25.1	16.3	0.9
*sec*-Butyl isothiocyanate	7.3	3.8	8.4	8.7	3.7	6.3
*(Z)*-3-Hexenal	5.1	1.5	0.7	2.4	3.8	2.0
3-(Methylthio)propyl isothiocyanate	0.9	0.8	4.0	1.8	4.6	2.2
Benzyl isothiocyanate	1.3	2.1	0.8	1.0	1.5	1.5

*The odorants are listed according to their average content, starting with the highest value*.

**Table 3:** Content [ng/g] of important aroma-active compounds in one gram of freshly ground horseradish root of six different horseradish varieties, determined by HRGC-MS or HRGC-GC-MS/O.

**Odorant**	**Content [ng/g]**					
	**VFS × BA**	**BA × VFS**	**BA**	**Kroener**	**Nyehemes**	**NA**
3-Isopropyl-2-methoxypyrazine^a^	21.5	13.3	10.3	6.8	95.2	30.8
3-*sec*-Butyl-2-methoxypyrazine^a^	11.5	8.5	32.2	26.9	58.1	6.7
3-Methylindole (skatole)^a^	5.9	1.3	10.3	13.2	3.2	8.5
(3*S*,3a*S*,7a*R*)-Wine lactone^b^	10.2	7.1	9.6	1.0	3.8	1.6

*The odorants are listed according to their average content, starting with the highest value*.

a *Content determined by HRGC-MS*.

b *Content determined by HRGC-GC-MS/O*.

Overall, all varieties showed comparable contents for AITC, with the sole exception of variety BA × VFS that contained less than 1,000 μg AITC/g. With regard to PEITC, variety VFS × BA yielded the lowest and Kroener the second lowest content, both with values below 100 μg/g. In case of CETP, the varieties were clustered into two groups, with varieties BA × VFS and NA displaying contents below 1 μg/g whereas the other varieties contained amounts above 15 μg/g. Apart from that, the *sec*-butyl ITC and benzyl ITC were quite comparable with regard to concentration in the different varieties, with concentrations of about 6 and 1 μg/g, respectively. The same was true for skatole and (3*S*,3a*S*,7a*R*)-wine lactone with very low values in the range of 1.3–13.2 and 1.0–10.2 ng/g, respectively. In contrast to that, *(Z)*-3-hexenal concentrations stood out in case of the variety VFS x BA with a content of 5.1 μg/g, while a relatively pronounced concentration of 4.6 μg/g was observed for 3-(methylthio)propyl ITC in case of variety Nyehemes. Moreover, the variety Nyehemes additionally displayed by far the highest contents of 3-isopropyl-2-methoxypyrazine (95.2 μg/g) and 3-*sec*-butyl-2-methoxypyrazine (58.1 μg/g).

## Discussion

This study demonstrates that different horseradish varieties show differences in their aroma profile even when grown under equal conditions.

Thereby, all varieties showed, in general, the same main aroma compounds, however, with some divergence between varieties with respect to FD factors and, accordingly, the corresponding contents of the respective aroma compounds. Ranked according to their FD factors starting with the highest, these substances were: 3-isopropyl-2-methoxypyrazine, AITC, (3*S*,3a*S*,7a*R*)-wine lactone, PEITC, *(Z)*-3-hexenal, *sec*-butyl-2-methoxypyrazine, skatole, the unknown compound at RI 1011, 3-(methylthio)propanal, 3-(methylthio)propyl ITC, CETP, benzyl ITC, and *sec*-butyl ITC. These results confirm our previous findings (Kroener and Buettner, 2017), with only some slight variations in the respective order of these odorous substances, according to average FD factors, in the different varieties.

Comparison of the FD factors and the corresponding semi-quantitative data reveals a good correlation, with varieties containing higher amounts of a certain substance generally also showing higher FD factors for the respective substances. This demonstrates that AEDA provides semi-quantitative indications via the relative ratios of the respective FD factors for specific substances, with some common variation of one or maximum two dilution steps due to natural variances in olfactory performance (Buettner and Schieberle, 2001).

In comparison to the other varieties, variety VFS × BA revealed both an increased FD factor by two dilution steps and a higher content in freshly ground root material for *(Z)*-3-hexenal, which is formed from linoleic acid through lipid oxidation (MacLeod, 1976), as well as a decreased FD factor for PEITC, comprising on average three dilution steps. On the other hand we observed a slightly decreased FD factor for *(Z)*-3-hexenal for variety BA by two dilution steps. Variety Nyehemes showed slightly higher FD factors and contents for 3-isopropyl-2-methoxypyrazine and 3-*sec*-butyl-2-methoxypyrazine. The FD factors were increased in both cases by, on average, two dilution steps. Especially the content for 3-isopropyl-2-methoxypyrazine clearly differed from those of the other varieties (cf. Table 3), showing a 6-fold higher value than the average value of the other varieties. An increased FD factor of, on average, three dilution steps for PEITC was detected for variety NA.

A comparison of these findings with those of our previous study (Kroener and Buettner, 2017) shows that only the slightly increased FD factors for the pyrazines in variety Nyehemes are in accordance with our previous study, while the other substances showed only slight differences in FD factors between the different varieties. This might be due to the fact that the varieties were grown on different fields and, accordingly, under different growing conditions.

As mentioned before the concentrations of CETP tended to two extremes in the investigated varieties. CETP was either detected with an FD factor of 16/32, or was olfactorily not perceivable at all, most likely due to concentrations below its odor threshold (cf. Table 1). When comparing these results with those of our previous study (Kroener and Buettner, 2017), only the data for variety Kroener, BA × VFS and NA agree with our previous reports. This indicates that variety Kroener is commonly characterized by a relatively high content for CETP, and that varieties BA × VFS and NA exhibit rather low contents. In this respect, this might point into the direction that, with regards to CETP, soil composition and climatic conditions do not so strongly impact the contents of CETP in those varieties. However, this observation would need to be further substantiated by more extended investigations comprising several consecutive cultivation seasons under different soil conditions.

With regards to odorant identification, we provide here the first unequivocal identification of (3*S*,3a*S*,7a*R*)-wine lactone in horseradish roots via mass spectrometric verification (cf. Figure 2). We further identified three previously in horseradish unknown substances by matching RI values and sensory characteristics with those of authentic reference standards. One of them is sotolone, which has been reported in lovage extract (Blank and Schieberle, 1993) and in fenugreek (Blank et al., 1997). The source for this compound in horseradish is unclear; it could be either endogenously generated through biosynthesis by the root itself or absorbed by the horseradish root from surrounding soil; in view of this it is interesting to note that sotolone may indeed stem from different biogenous sources, as it has also been identified in aquaculture water (Mahmoud and Buettner, 2016). The second newly discovered compound is 3-isobutyl-2-methoxypyrazine, which has already been described in wasabi (Imazaki, 2012), peas and lettuce (Murray and Whitfield, 1975). The formation of methoxypyrazines in plants has been reported to take place by amidation and condensation of an α-amino acid and an unknown 1,2-dicarbonyl compound to a 2-hydroxy-3-alkylpyrazine that is further methylated by an *O*-methyltransferase (Wuest, 2017). The last new substance was *p*-anisaldehyde, which has been reported in wild fennel (Piccaglia and Marotti, 2001), fennel seeds (Marotti et al., 1993), and aniseed (Orav et al., 2008), and is probably biosynthesized by plants via the phenylpropanoid pathway (Kundu and Mitra, 2016).

It is important to note that the varieties of the present study were cultivated on a different acreage than in our previous investigation (Kroener and Buettner, 2017). This could be one of the reasons, why we found new substances in the same varieties; on the other hand, climatic differences between the different cultivation years might also account for these differences. This together with some minor modifications in the sample work-up might be the reason why some aroma-active compounds could not be detected in the present study. These were butyl ITC, *(Z)*-3-hexen-1-ol, 1-nonen-3-one, *(Z)*-1,5-octadien-3-one, 2-phenylethanol, *(E)*-1,5-undecadien-3-ol and the group of methylthioalkyl ITCs comprising a chain length from four to seven carbon atoms in the alkyl moiety.

Several other studies reported contents of AITC and PEITC in horseradish roots ranging from 700 to 3,300 μg/g and from 55 to 541 μg/g fresh weight (FW) (Masuda et al., 1996; Sultana et al., 2003; D'auria et al., 2004; Horbowicz and Rogowska, 2006; Kosson and Horbowicz, 2008; Kübler, 2010). These values are in good agreement with our data for AITC (936–1,990 μg/g) and PEITC (approximately 40–270 μg/g). However, the lowest value for PEITC of about 40 μg/g in our study is slightly lower than the minimal value of 55 μg/g reported by Horbowicz et al.

Ku et al. (2015) determined contents of CETP in horseradish roots from 0.00 to 11.4 μmol/g dry weight (DW), which correspond to approximately 0–1,130 μg/g DW and to 0.00–283 μg/g FW, when calculating with a DW of 25% (Scherz and Senser, 2000). The content range in our study was 0.2–25.1 μg/g, which is clearly lower than that reported by Ku et al. It is surprising that about 70% of the values for CETP found by Ku et al. were higher than about 40 μg/g FW, whereas our highest value for this compound was 25.1 μg/g only. This difference might be due to the fact that the horseradish samples analyzed in both studies belong to different varieties and were grown under different conditions—amongst others a different growth year, climatic conditions, and different types of soil. Differences in sample preparation and chemical analysis between both studies might be further influencing factors, as in the study by Ku et al. the extraction of freeze-dried horseradish samples was performed with a hexane-water mixture for 24 h and the quantification of CETP was conducted according to the effective carbon number concept. Apart from that it is well conceivable that particle size and fermentation time may strongly impact enzymatic formation of these compounds.

*sec*-Butyl ITC has been reported in horseradish with 2.77 ± 0.24 μg/g (Sultana et al., 2003) and 27 μg/g (Masuda et al., 1996), with close agreement of the data of Sultana et al. with our data, ranging from 3.7 to 8.7 μg/g, whereas the data reported by Masuda et al. is more than three times higher than the highest value of *sec*-butyl ITC determined in our study. To the best of our knowledge, the study by Masuda et al. is the only one that reports quantitative data for benzyl ITC and 3-(methylthio)propyl ITC in horseradish roots. Regarding benzyl ITC, the authors determined a content of 1.6 μg/g that matches very well with our data in the range from 0.8 to 2.1 μg/g. Their reported value of 1.5 μg/g for 3-(methylthio)propyl ITC likewise shows good correlation with our content range of this substance from 0.8 to 4.6 μg/g.

We deliberately set a sensory analysis of the different varieties aside, as the change in aroma composition of freshly ground horseradish is very dynamic and takes place in a relatively short time period, especially in regard to the ITCs, as we have shown in our previous study (Kroener and Buettner, 2017).

## Conclusion

With our study we successfully revealed those substances that generally represent the most important aroma-active compounds in horseradish roots, and were able to elaborate characteristics of specific horseradish varieties, such as relatively high FD factors and contents of 3-isopropyl-2-methoxypyrazine and 3-*sec*-butyl-2-methoxypyrazine for variety Nyehemes, when compared to other varieties. Additionally we approximated the concentration levels for most of these compounds via semi-quantitative analysis. To the best of our knowledge, we report here for the first time semi-quantitative data for *(Z)*-3-hexenal, 3-isopropyl-2-methoxypyrazine, 3-*sec*-butyl-2-methoxypyrazine, skatole and (3*S*,3a*S*,7a*R*)-wine lactone in horseradish roots that need to be, however, substantiated by future additional quantification experiments. Our data, however, reveal that most of these substances are present in horseradish in very low amounts in the ng/g area, yet eliciting some of the highest FD factors; this again demonstrates that absolute concentration alone does not allow for any conclusion about the actual odor potency of a specific substance in the respective food, and that the odor potency of the substance needs to be taken into consideration. Accordingly, this study provides the necessary basis for future studies that investigate the aroma of horseradish and its different varieties.

## Author contributions

All authors listed have made a substantial, direct and intellectual contribution to the work, and approved it for publication.

### Conflict of interest statement

The authors declare that the research was conducted in the absence of any commercial or financial relationships that could be construed as a potential conflict of interest.
